# TGFβ-Stimulated MicroRNA-21 Utilizes PTEN to Orchestrate AKT/mTORC1 Signaling for Mesangial Cell Hypertrophy and Matrix Expansion

**DOI:** 10.1371/journal.pone.0042316

**Published:** 2012-08-03

**Authors:** Nirmalya Dey, Nandini Ghosh-Choudhury, Balakuntalam S. Kasinath, Goutam Ghosh Choudhury

**Affiliations:** 1 Department of Medicine, University of Texas Health Science Center at San Antonio, San Antonio, Texas, United States of America; 2 Department of Pathology, University of Texas Health Science Center at San Antonio, San Antonio, Texas, United States of America; 3 Geriatric Research, Education and Clinical Center, South Texas Veterans Health Care System, San Antonio, Texas, United States of America; 4 Veterans Administration Research, South Texas Veterans Health Care System, San Antonio, Texas, United States of America; National Center for Scientific Research Demokritos, Greece

## Abstract

Transforming growth factor-β (TGFβ) promotes glomerular hypertrophy and matrix expansion, leading to glomerulosclerosis. MicroRNAs are well suited to promote fibrosis because they can repress gene expression, which negatively regulate the fibrotic process. Recent cellular and animal studies have revealed enhanced expression of microRNA, miR-21, in renal cells in response to TGFβ. Specific miR-21 targets downstream of TGFβ receptor activation that control cell hypertrophy and matrix protein expression have not been studied. Using 3′UTR-driven luciferase reporter, we identified the tumor suppressor protein PTEN as a target of TGFβ-stimulated miR-21 in glomerular mesangial cells. Expression of miR-21 Sponge, which quenches endogenous miR-21 levels, reversed TGFβ-induced suppression of PTEN. Additionally, miR-21 Sponge inhibited TGFβ-stimulated phosphorylation of Akt kinase, resulting in attenuation of phosphorylation of its substrate GSK3β. Tuberin and PRAS40, two other Akt substrates, and endogenous inhibitors of mTORC1, regulate mesangial cell hypertrophy. Neutralization of endogenous miR-21 abrogated TGFβ-stimulated phosphorylation of tuberin and PRAS40, leading to inhibition of phosphorylation of S6 kinase, mTOR and 4EBP-1. Moreover, downregulation of miR-21 significantly suppressed TGFβ-induced protein synthesis and hypertrophy, which were reversed by siRNA-targeted inhibition of PTEN expression. Similarly, expression of constitutively active Akt kinase reversed the miR-21 Sponge-mediated inhibition of TGFβ-induced protein synthesis and hypertrophy. Furthermore, expression of constitutively active mTORC1 prevented the miR-21 Sponge-induced suppression of mesangial cell protein synthesis and hypertrophy by TGFβ. Finally, we show that miR-21 Sponge inhibited TGFβ-stimulated fibronectin and collagen expression. Suppression of PTEN expression and expression of both constitutively active Akt kinase and mTORC1 independently reversed this miR-21-mediated inhibition of TGFβ-induced fibronectin and collagen expression. Our results uncover an essential role of TGFβ-induced expression of miR-21, which targets PTEN to initiate a non-canonical signaling circuit involving Akt/mTORC1 axis for mesangial cell hypertrophy and matrix protein synthesis.

## Introduction

Accumulation of extracellular matrix in chronic kidney disease is preceded by renal hypertrophy especially glomerular mesangial hypertrophy. Mesangial cell among the three cell types in the glomerulus acts as the predominant site for the synthesis of extracellular matrix proteins, which contribute to glomerular hypertrophy and renal fibrosis found in progressive chronic kidney diseases [1]. Various growth factors and cytokines produced by the infiltrating cells during the disease process and by the local kidney cells participate in the fibrotic process [2]. Among these, TGFβ produced by the kidney cells and by the infiltrating macrophages plays a significant role in the pathogenesis of mesangial matrix expansion [3]. Increased glomerular expression of TGFβ has been reported in both experimental and human kidney disease [3], [4]. Mice with increased plasma TGFβ1 levels displayed enhanced renal fibrosis [5]. On the other hand, blockage of TGFβ1 prevented renal especially glomerular hypertrophy and fibrosis in mouse with diabetes [6], [7].

TGFβ initiates its signal transduction by binding to the type II receptor, which forms the oligomeric complex containing the type I receptor. In the tetrameric receptor complex, type II receptor phosphorylates type I receptor in the GS domain, which releases FKBP12 from the receptor, resulting in activation of the type I receptor serine threonine kinase. L45 loop of receptor kinase domain located immediately downstream of the GS segment interacts with the L3 loop of receptor-specific Smad 3 and 2 followed by phosphorylation of serine residues in the C-terminus of Smad protein [8], [9]. This binding of the receptor to Smads is also facilitated by SARA, a FYVE domain containing protein, which immobilizes receptor-specific Smads to the plasma membrane [10]. Phosphorylated Smad dissociates from the receptor resulting in exposure of the nuclear import sequence and heterodimerization with the common Smad, Smad 4. The heteromeric Smad complex then translocates to the nucleus, recruits transcriptional co-activators or co-repressors and regulates target gene expression [9], [11], [12]. Both in human and animal models of kidney fibrosis, TGFβ-specific Smads are activated, which increases transcription of various collagens [13]. Deletion of Smad 3 in mice protects from fibrotic disorders of kidney [14], [15], [16]. Although both Smad 3 and Smad 2 act downstream of TGFβ, unexpectedly, specific deletion of Smad 2 in kidney significantly enhanced Smad 3 activity, collagen matrix expansion and fibrosis, indicating that Smad 2 functions as a negative regulator of TGFβ-driven renal fibrosis [17]. Along with this canonical signal transduction pathway, TGFβ stimulates non-canonical signaling which includes activation of the tyrosine and serine threonine kinases, such as c-Src, Erk1/2, JNK and p38 MAP kinases [18], [19], [20]. Also, TGFβ activates PI 3 kinase/Akt signaling [21], [22]. More recently we and others have shown that TGFβ regulates PI 3 kinase-dependent mTOR to increase cellular hypertrophy including mesangial cell hypertrophy [23], [24], [25].

miRNAs regulate expression of genes via post-transcriptional mechanism [26]. miRNAs are transcribed by RNA polymerase II similar to mRNAs and contain a 5′ CAP and a 3′ poly A tail [26], [27], [28]. In the nucleus primary transcripts of the miRNAs are processed by *Drosha* RNase III activity to produce stem-loop containing pre-miR, which are exported to the cytoplasm and further processed by *Dicer* in a complex containing TRBP. Recently, MCPIP1, an antagonist of *Dicer*, has been shown to act on the terminal loop of pre-miR to block *Dicer* activity [29]. Thus a concerted action of both these proteins produces ∼ 22 nucleotide long double stranded RNAs. The miRNA guide strand is produced from this duplex, which then binds to Argonaute 2 in the RISC to interact with the specific miRNA recognition element present in the 3′ UTR of target mRNAs [26], [27]. This binding destabilizes the target mRNA and predominantly suppresses the translation of mRNA although degradation of mRNAs can also occur [26], [27], [28]. Conditional deletion of *Dicer* in the nephron progenitors showed that global loss of miRNAs induced a significant loss of nephron number [30], [31]. Deletion of either *Dicer* or *Drosha* in the mouse glomerular podocytes showed loss of renal function with glomerulosclerosis, foot process effacement and proteinuria [31], [32], [33], [34]. Interestingly, deletion of *Dicer* in proximal tubules protected mice from ischemia reperfusion injury [35]. The expression of myriad of miRNAs is altered in various kidney diseases [13], [36], [37], [38]. Recently, a role of miR-21 in diabetes-induced pancreatic β cell death has been demonstrated [39]. Also, TGFβ-mediated increase in miR-21 levels has been linked to the progression of disease in mouse models of fibrosis [40], [41], [42]. In the proximal tubular epithelial cells, TGFβ-stimulated matrix protein expression was linked to expression of miR-21 [41]. However, the signaling pathway miR-21 utilizes for fibrotic protein expression is poorly understood. Here we demonstrate that TGFβ-stimulated expression of miR-21 in glomerular mesangial cells inhibits PTEN protein levels, which results in activation of Akt and mTORC1. Furthermore, we show that TGFβ forces miR-21-targeted PTEN to upregulate protein synthesis and hypertrophy that is controlled by Akt/TORC1 signaling. Finally, we depict that miR-21-induced increase in two fibrotic matrix proteins fibronectin and collagen I (α2) uses PTEN/Akt/TORC1 pathway.

## Results

### miR-21 Regulates PTEN-mediated Akt Activation in Human Glomerular Mesangial Cells

**Figure 1:**
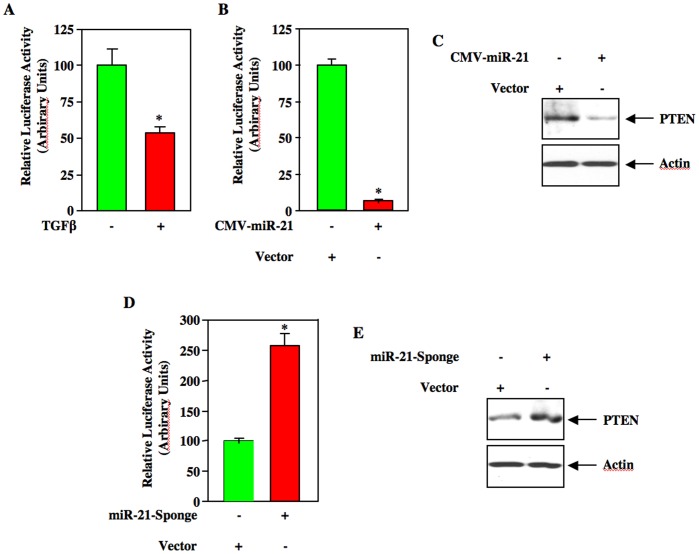
TGFβ-stimulated miR-21 targets PTEN 3′UTR to inhibit PTEN expression. (A) Human glomerular mesangial cells were transfected with PTEN 3′UTR-containing luciferase (PTEN 3′UTR-Luc) reporter plasmid PTEN 3′UTR-Luc. Transfected cells were serum-starved for 16 hours followed by incubation with 2 ng/ml TGFβ for 24 hours. The cell lysates were assayed for luciferase activity as described in the Materials and Methods [62], [82], [102]. Mean ± SE of six measurements is shown. *p = 0.018 vs control. (B and C) Mesangial cells were cotransfected with PTEN 3′UTR-Luc and CMV-miR-21 (expressing mature miR-21). The cell lysates were assayed for luciferase activity as described (panel B) [62], [82], [102]. Mean ± SE of triplicate measurements is shown; *p = 0.003 vs vector. For panel C, the cell lysates were immunoblotted with PTEN and actin antibodies. (D and E) Mesangial cells were transfected with PTEN 3′UTR-Luc plus miR-21 Sponge. For panel D the cell lysates were assayed for luciferase activity as described [62], [82], [102]. Mean ± SE of 12 measurements is shown; *p = 0.0001 vs vector. For panel E, the cell lysates were immunoblotted with PTEN and actin antibodies.

Recently, TGFβ has been shown to enhance the expression of miR-21 in rodent glomerular mesangial cells in culture; however, the signaling role of miR-21 in TGFβ-induced cellular hypertrophy and matrix protein expression has not been studied [40]. We confirmed the expression of mature miR-21 as well as pre-miR-21 in human mesangial cells (Fig. S1). The miR-21 target that mediates pathologic consequences of TGFβ has not been identified in renal cells. We and others have recently identified the tumor suppressor protein PTEN as a regulator of TGFβ-induced glomerular mesangial cell hypertrophy and matrix protein expression [25], [43]. The 3′UTR of PTEN mRNA has been experimentally validated as a target of miR-21 [44], [45]. Therefore, to initiate studies involving miR-21 and PTEN, we tested the effect of TGFβ on the reporter activity of a plasmid in which the firefly luciferase cDNA is fused to 3′UTR of PTEN (PTEN 3′UTR-Luc). Transient transfection assay using this plasmid in human mesangial cells showed significant repression of reporter activity in response to TGFβ (Fig. 1A). Since miR-21 is increased in TGFβ-stimulated mesangial cells (Fig. S1), we examined the effect of this miRNA. Plasmid-derived expression of miR-21 significantly inhibited the reporter activity of PTEN 3′UTR-Luc (Fig. 1B, Fig. S2A). Also, expression of miR-21 suppressed PTEN protein levels (Fig. 1C and Fig. S2B) To confirm the role of miR-21, we used a plasmid vector called ‘miR-21 Sponge’, which contains 7 copies of bulged miR-21 binding site fused to the 3′ end of GFP mRNA (Fig. S3A). Expression of this construct neutralizes miR-21 in cells [46]. Human mesangial cells were transiently transfected with PTEN 3′UTR-Luc and miR-21 Sponge. Expression of miR-21 Sponge significantly increased the luciferase activity (Fig. 1D). Concomitantly, miR-21 Sponge increased PTEN protein expression (Fig. 1E). Expression of GFP mRNA was used as a surrogate for miR-21 Sponge expression (Fig. S3B and S3C). These results suggest that miR-21 targets PTEN in mesangial cells.

**Figure 2:**
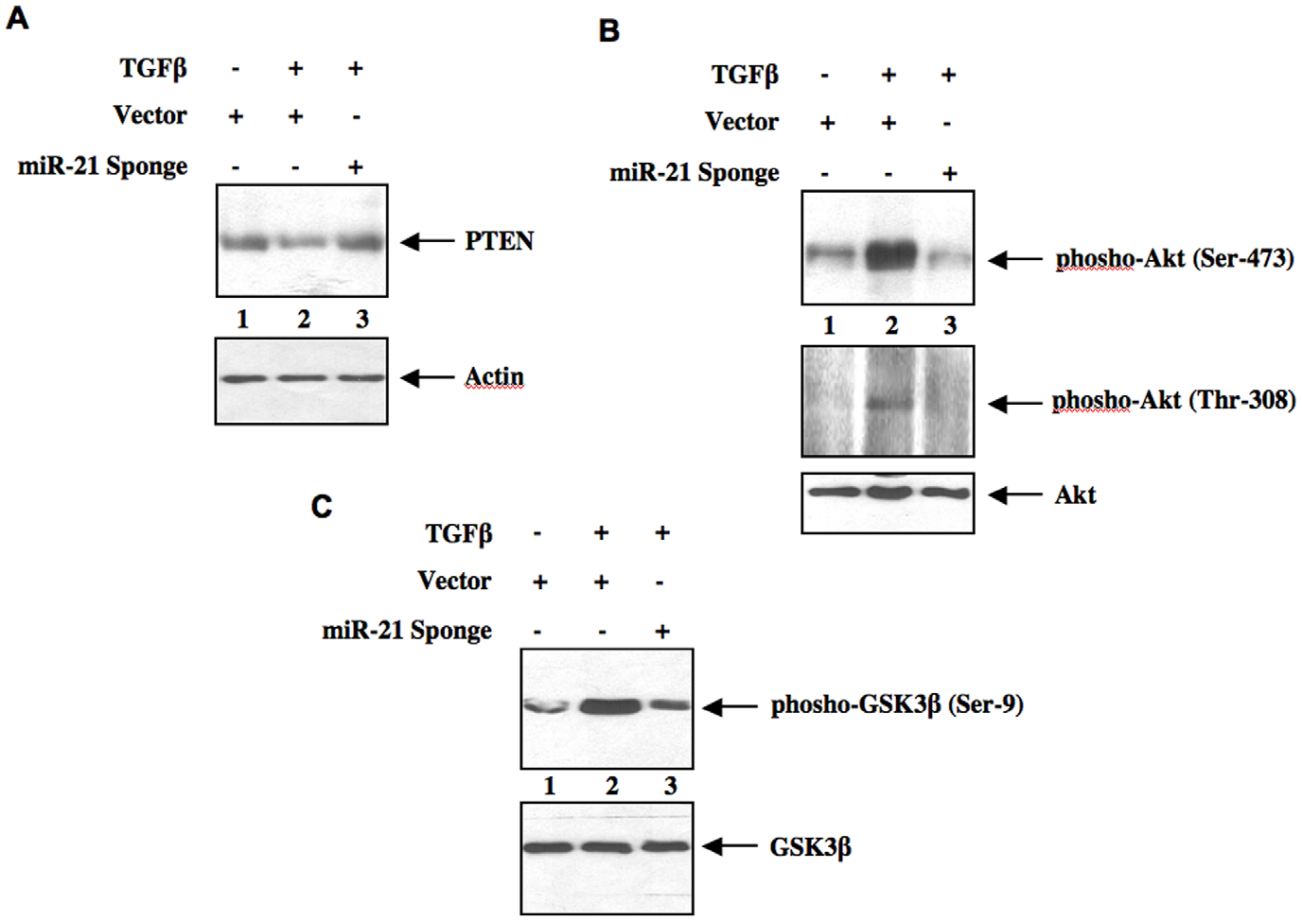
miR-21 targets PTEN to regulate activation of Akt kinase in response to TGFβ. Mesangial cells were transfected with miR-21 Sponge or vector followed by incubation with 2 ng/ml TGFβ for 24 hours. Cell lysates were immunoblotted with PTEN, actin (panel A), phospho-Akt (Ser-473), phospho-Akt (Thr-308), Akt (panel B), phospho-GSK3β and GSK3β (panel C) antibodies as indicated.

To elucidate the role of miR-21-in TGFβ-mediated signal transduction, we used miR-21 Sponge. As expected TGFβ inhibited the expression of PTEN protein in mesangial cells (Fig. 2A) [25], [43]. Expression of miR-21 Sponge significantly prevented TGFβ-mediated decrease in PTEN levels (Fig. 2A and Fig. S4A). PTEN is an endogenous inhibitor of PI 3 kinase-dependent Akt activation [47], [48]; it inhibits phosphorylation of Akt as we have previously shown in mesangial cells [25]. Thus, downregulation of PTEN in response to TGFβ increased phosphorylation of Akt at both catalytic loop and hydrophobic motifs (Fig. 2B). Expression of miR-21 Sponge blocked TGFβ-stimulated phosphorylation of Akt (Fig. 2B and Fig. S4B). Since both these phosphorylations of Akt regulate its enzymatic activity, we tested the phosphorylation of one of the endogenous substrates of this kinase, GSK3β as an index of Akt kinase activity. Concomitant with Akt phosphorylation, TGFβ increased phosphorylation of GSK3β, which was prevented by expression of miR-21 Sponge (Fig. 2C and Fig. S4C).

### TGFβ-stimulated mTORC1 Activation is Mediated by miR- 21

**Figure 3:**
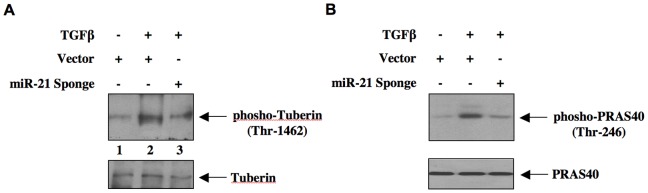
miR-21 Sponge inhibits TGFβ-stimulated phosphorylations of tuberin and PRAS40. Glomerular mesangial cells were transfected with miR-21 Sponge or vector. The serum-starved cells were incubated with 2 ng/ml TGFβ for 24 hours. The cell lysates were immunoblotted with phospho-tuberin (Thr-1462), tuberin (panel A), phospho-PRAS40 (Thr-246) and PRAS40 (panel B) antibodies as indicated.

**Figure 4:**
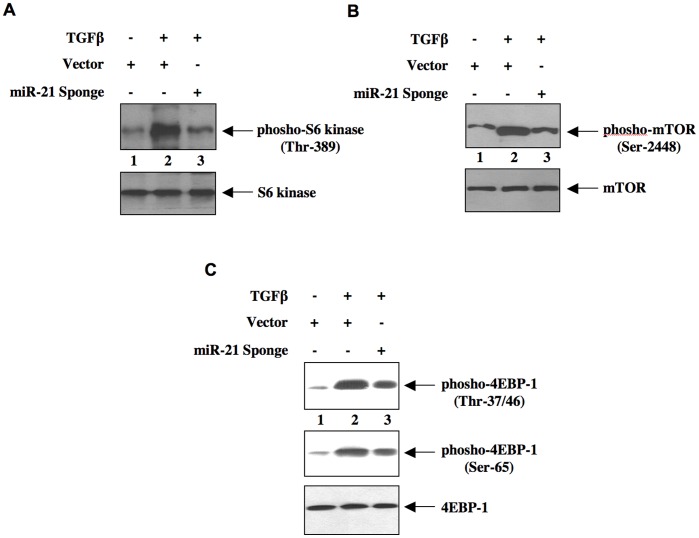
miR-21 Sponge blocks mTORC1 activity in response to TGFβ. Mesangial cells were transfected with miR-21 Sponge or vector. The serum-starved cells were incubated with 2 ng/ml TGFβ for 24 hours. The cell lysates were immunoblotted with phospho-S6 kinase (Thr-389), S6 kinase (panel A), phospho-mTOR (Ser-2448), mTOR (panel B), phospho-4EBP-1 (Thr-34/46), phospho-4EBP-1 (Ser-65) and 4EBP-1 (panel C) antibodies as indicated.

We and others have recently shown activation of mTOR by TGFβ [23], [24]. Furthermore, role of mTORC1 has been established in rodent models of fibrosis where TGFβ plays important role [49], [50], [51], [52]. Therefore, we tested the effect of miR-21-driven PTEN expression in TGFβ-stimulated mTORC1 activation. Two proteins, tuberin and PRAS40, act as suppressors of mTORC1 activity [23], [53], [54], [55]. Phosphorylation of both these proteins by Akt inactivates them, resulting in activation of mTORC1 [53], [54], [56]. We first examined the role of miR-21 in TGFβ-induced phosphorylation of tuberin. Expression of miR-21 Sponge inhibited TGFβ-stimulated phosphorylation of tuberin (Fig. 3A and Fig. S5A). Similarly, phosphorylation of PRAS40 by TGFβ was blocked by expression of miR-21 Sponge (Fig. 3B and Fig. S5B). Next, we determined activation of mTORC1. We used Thr-389 phosphorylation of S6 kinase, which is a known substrate of mTORC1, as a surrogate for mTORC1 activation [57]. TGFβ increased phosphorylation of S6 kinase (Fig. 4A). Expression of miR-21 Sponge inhibited TGFβ-induced phosphorylation of S6 kinase (Fig. 4A and Fig. S6A). Recently, it has been shown that activated S6 kinase phosphorylates mTOR at Ser-2448 [58]. Therefore, we examined the effect of mir-21 Sponge on phosphorylation of mTOR. Expression of miR-21 Sponge attenuated TGFβ-stimulated phosphorylation of mTOR (Fig. 4B and Fig. S6B). Activated mTORC1 phosphorylates the mRNA translation initiation factors. One such protein is 4EBP-1, which undergoes phosphorylation at Thr-37/46 and Ser-65 residues [59]. TGFβ increased phosphorylation at all these residues (Fig. 4C). Expression of miR-21 Sponge inhibited phosphorylation of 4EBP-1 at these sites (Fig. 4C and Fig. S6C). These results indicate that miR-21 regulates TGFβ-induced mTORC1 activation in mesangial cells.

### miR-21 Regulates TGFβ-stimulated Mesangial Cell Hypertrophy by PTEN/Akt/mTORC1 Signal Transduction

**Figure 5:**
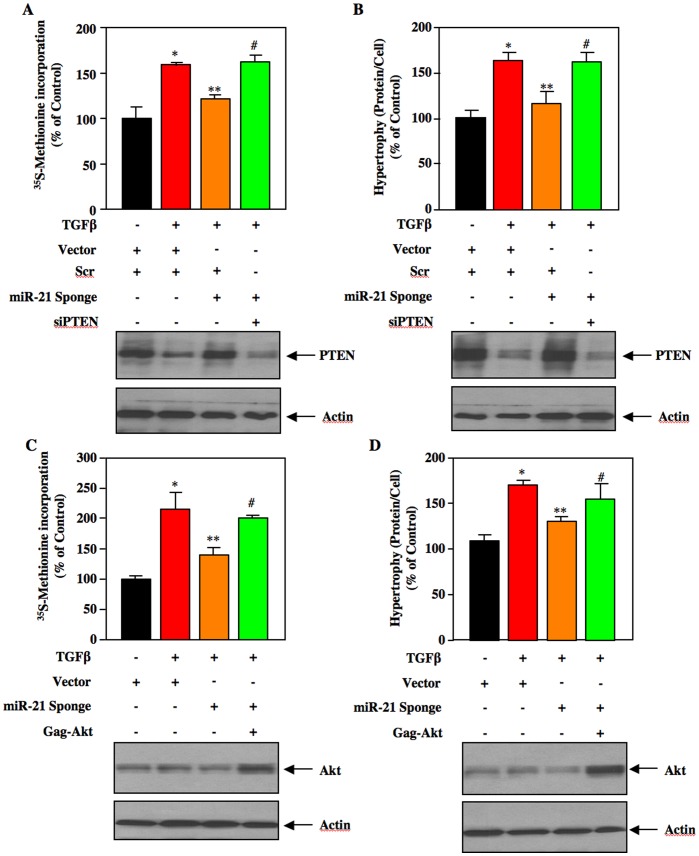
miR-21/PTEN/Akt axis regulates mesangial cell protein synthesis and hypertrophy in response to TGFβ. Mesangial cells were cotransfected with miR-21 Sponge and siRNAs targeting PTEN mRNA (siPTEN) or scrambled RNA (Scr) (panels A and B). Mesangial cells were cotransfected with miR-21 Sponge and constitutively active Gag-Akt as indicated (panels C and D). The transfected cells were starved for 16 hours prior to incubation with 2 ng/ml TGFβ for 24 hours. Protein synthesis (panels A and C) and hypertrophy (panels B and D) were determined as described in the Materials and Methods [23], [25], [55]. Mean ± SE of 3 measurements is shown. For panel A, *p<0.01 vs control; **p<0.01 vs TGFβ; #p<0.05 vs miR-21 Sponge plus TGFβ. For panel B, *p<0.05 vs control; **p<0.05 vs TGFβ; #p<0.05 vs miR-21 Sponge plus TGFβ. For panel C, *p<0.05 vs control; **p<0.05 vs TGFβ; #p<0.05 vs miR-21 Sponge plus TGFβ. For panel D, *p<0.01 vs control; **p<0.05 vs TGFβ; #p<0.05 vs miR-21 Sponge plus TGFβ. Bottom panels show expression of PTEN and Akt in representative samples. Actin expression was used as a control for immunoblotting.

We and others have recently reported a role of PTEN in mesangial cell hypertrophy. Furthermore, TGFβ promoted protein synthesis and mesangial cell hypertrophy [23], [25], [43]. We tested the involvement of miR-21-targeted PTEN in this process. As expected, TGFβ increased protein synthesis due to reduced PTEN expression (Fig. 5A) [25]. Expression of miR-21 Sponge reversed TGFβ-inhibited PTEN expression (Fig. 2A) and significantly attenuated TGFβ-induced protein synthesis (Fig. 5A and Fig. S7A). To specifically investigate the requirement of PTEN for the effect of miR-21 Sponge, we used siRNAs against PTEN mRNA. Expression of PTEN siRNAs significantly reversed the miR-21 Sponge-mediated inhibition of TGFβ-induced protein synthesis (Fig. 5A and Fig. S7A). Similarly, expression of miR-21 Sponge significantly inhibited TGFβ-stimulated hypertrophy of mesangial cells (Fig. 5B and Fig. S7B). Downregulation of PTEN using siRNAs markedly prevented the inhibitory effect of miR-21 Sponge on TGFβ-induced hypertrophy (Fig. 5B and Fig. S7B). Next, we determined whether the miR-21-targeted PTEN uses Akt kinase for these processes. We used a plasmid vector containing Gag-Akt, which behaves as a constitutively active kinase [60]. Expression of Gag-Akt significantly reversed the inhibitory effect of miR-21 Sponge on TGFβ-stimulated protein synthesis and hypertrophy (Fig. 5C and 5D; Fig. S7C and S7D). These results suggest that TGFβ-induced expression of miR-21 uses PTEN/Akt signaling in regulating mesangial cell protein synthesis necessary for hypertrophy.

**Figure 6:**
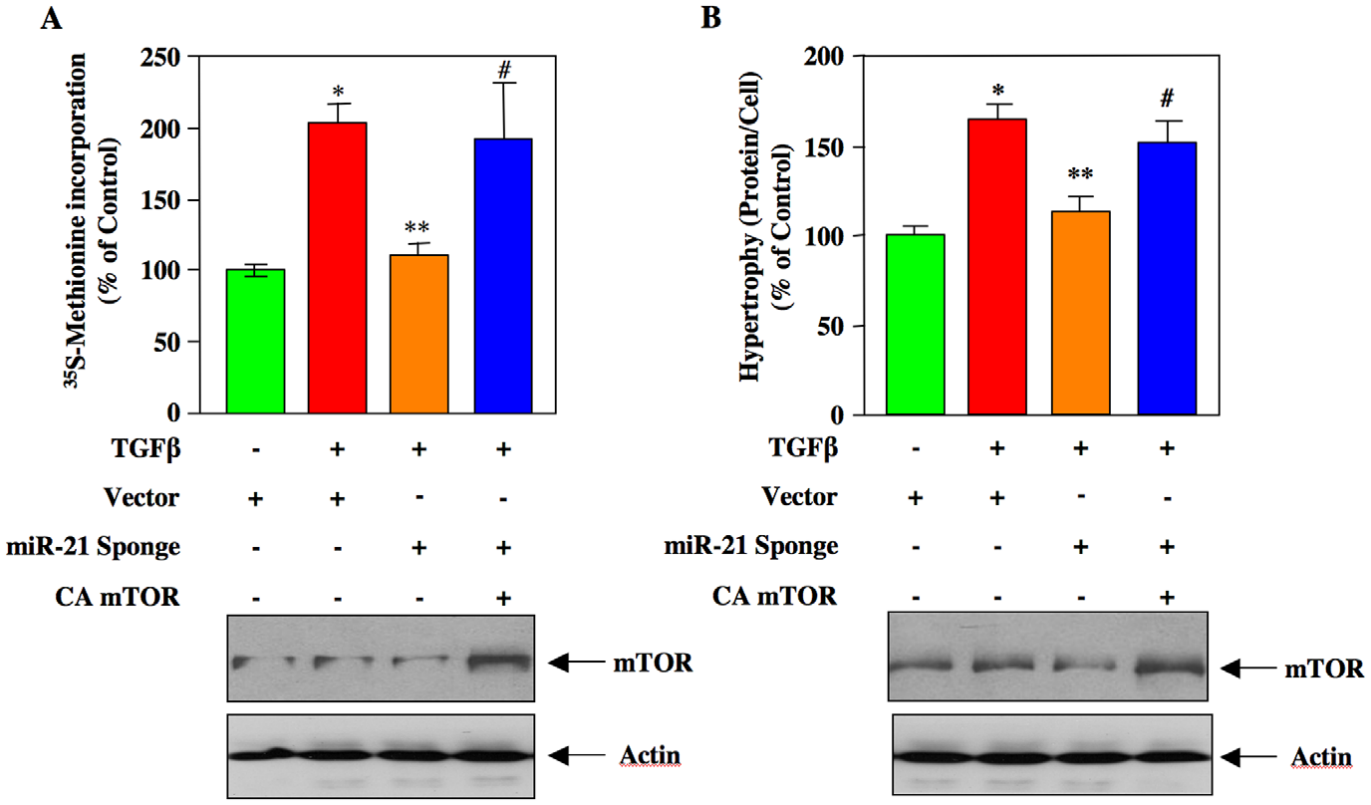
Expression of constitutively active mTORC1 blocks the inhibitory effect of miR-21 Sponge on TGFβ-induced mesangial cell protein synthesis and hypertrophy. Glomerular mesangial cells were cotransfected with miR-21 Sponge and CA mTOR as indicated. The cells were incubated with 2 ng/ml TGFβ for 24 hours. Protein synthesis (panel A) and hypertrophy (panel B) were determined as described [23], [25], [55]. For panel A, mean ± SE of triplicate measurements is shown; *p<0.05 vs control; **p<0.05 vs TGFβ; #p<0.05 vs miR-21 Sponge in the presence of TGFβ. For panel B, mean ± SE of triplicate measurements is shown; p<0.01 vs control; **p<0.01 vs TGFβ; #p<0.05 vs miR-21 Sponge in the presence of TGFβ. Bottom panels show expression of mTOR and actin in the representative samples.

The results described in Figure 4 demonstrate that miR-21 regulates mTORC1 activity, which phosphorylates 4EBP-1. mTORC1-mediated phosphorylation of 4EBP-1 results in its inactivation and initiation of protein synthesis, necessary for cellular hypertrophy including mesangial cell hypertrophy [23], [55], [57], [59]. Therefore, we tested the role of mTORC1 in the action of miR-21 in TGFβ-induced protein synthesis. We used a vector expressing a mutant mTOR that has constitutive mTORC1 activity [61], [62]. Expression of the constitutively active (CA) mTOR along with miR-21 Sponge significantly reversed the suppressive effect of miR-21 Sponge on both TGFβ-induced protein synthesis and hypertrophy of mesangial cells (Fig. 6A and 6B; Fig. S8A and S8B). Thus, our results demonstrate involvement of mTORC1 in the action of miR-21 in mesangial cell hypertrophy.

### miR-21 uses PTEN/Akt/mTORC1 Signaling for TGFβ-induced Matrix Protein Expression

**Figure 7:**
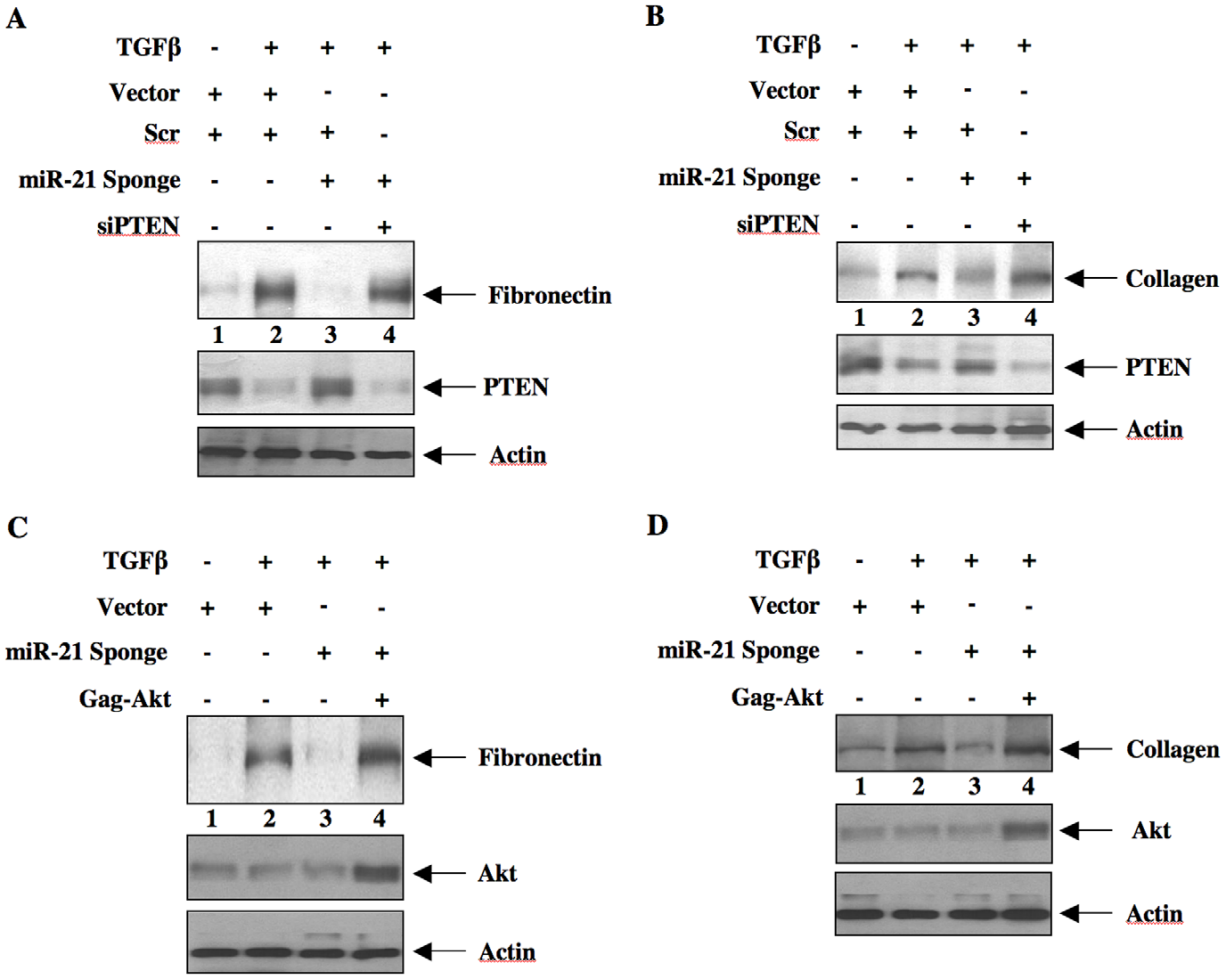
miR-21/PTEN/Akt axis regulates mesangial cell matrix protein expression in response to TGFβ. Mesangial cells were transfected with miR-21 Sponge and siPTEN or scrambled RNA as indicated in panels A and B. Similarly, mesangial cells were transfected with miR-21 Sponge and Gag-Akt as indicated in panels C and D. The transfected cells were incubated with 2 ng/ml TGFβ for 24 hours. The cell lysates were immunoblotted with fibronectin, PTEN, actin (panel A), collagen I (α2), PTEN, actin (panel B), fibronectin, Akt, actin (panel C) and collagen I (α2), Akt, actin (panel D) antibodies as indicated.

**Figure 8:**
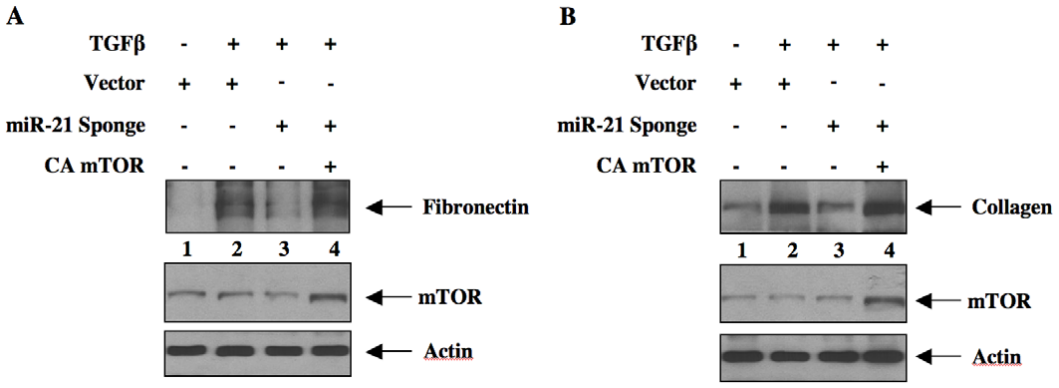
Constitutively active mTORC1 prevents the inhibition of miR-21 Sponge on TGFβ-stimulated fibronectin and collagen expression. Glomerular mesangial cells were transfected with miR-21 Sponge and CA mTOR plasmids as indicated. The cells were incubated with TGFβ for 24 hours. The cell lysates were immunoblotted with fibronectin (panel A) and collagen I (α2) (panel B) antibodies. Immunoblots of mTOR and actin are shown at the bottom.

TGFβ contributes to renal fibrosis by stimulating the synthesis of matrix proteins such as fibronectin and collagen I (α2) [3]. TGFβ stimulates expression of both these proteins in mesangial cells, which contribute to glomerulosclerosis [1]. We examined the role of miR-21-regulated PTEN in the expression of these proteins. As expected, incubation of mesangial cells with TGFβ increased the expression of fibronectin and collagen I (α2) (Fig. 7). Expression of miR-21 Sponge blocked both fibronectin and collagen I(α2) expression in response to TGFβ (Fig. 7 and Fig. S9). To test if PTEN is involved in this inhibition by miR-21 Sponge, we used siRNAs against PTEN. Downregulation of PTEN by siPTEN reversed the inhibition of both TGFβ-stimulated fibronectin and collagen expression by miR-21 Sponge (Fig. 7A and 7B and Fig. S9A and S9B). Since PTEN regulates activation of Akt kinase, we tested the involvement of Akt in miR-21 action. Expression of the constitutively active Gag-Akt prevented the miR-21 Sponge-induced suppression of TGFβ-stimulated fibronectin and collagen I (α2) expression (Fig. 7C and 7D and Fig. S9C and S9D). We have shown above that miR-21 controls TGFβ-stimulated mTORC1 activity. We examined the involvement of this kinase in fibronectin and collagen expression. Expression of CA mTOR, which acts as constitutively active mTORC1 [62], reversed the miR-21 Sponge-mediated inhibition of both fibronectin and collagen expression in response to TGFβ (Fig. 8A and 8B and Fig. S10A and S10B). Together these results indicate that miR-21-stimulated PTEN-Akt-mTORC1 signaling is required for TGFβ-stimulated fibrotic protein expression in mesangial cells.

## Discussion

**Figure 9:**
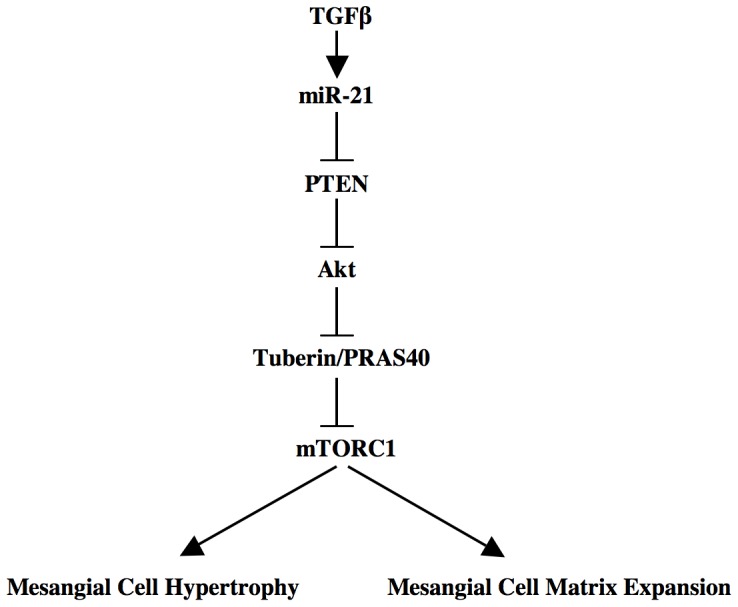
Schematic showing the results described in the paper. TGFβ-stimulated miR-21 decreases PTEN to activate Akt-dependent mTORC1, leading to hypertrophy of mesangial cells and matrix protein expression.

We demonstrate that increased miR-21 uses the tumor suppressor protein PTEN as its downstream target to regulate Akt/mTORC1 signaling in response to TGFβ in renal mesangial cells. We show that miR-21-targeted PTEN regulates TGFβ-induced protein synthesis required for mesangial cell hypertrophy. miR-21-stimulated Akt/mTORC1 cascade forces expression of two fibrotic proteins, fibronectin and collagen, for induction of glomerulosclerosis (Fig. 9).

Recently we and others have shown a role of PTEN in TGFβ-forced diabetic renal glomerular hypertrophy in rats and mouse [25], [43]. Downregulation of PTEN in renal glomeruli and in cultured mesangial cells in response to TGFβ contributes to renal hypertrophy and matrix protein expression [25], [43]. Abundance of PTEN is regulated at the levels of transcription, ubiquitination, phosphorylation, protein-protein interaction and oxidation [63]. More recently post-transcriptional control of expression of PTEN involving specific miRNAs has been elucidated. The miRNA, miR-192, regulates the expression of miR-216a and miR-217, both of which directly target the PTEN 3′UTR [43]. Upregulation of these three miRNAs has been reported in renal glomeruli of diabetic mice and in mesangial cells incubated with high glucose or TGFβ [43], [64]. They induced mesangial cell hypertrophy and matrix protein collagen expression. In contrast to this observation, a recent study demonstrated decreased expression of miR-192 in mesangial and proximal tubular epithelial cells and in mouse diabetic kidney cortex [65]. This observation was confirmed in a human study where a microRNA expression profiling in renal biopsies of patients with established diabetic nephropathy showed significantly reduced expression of miR-192, which correlated with low glomerular filtration rate and tubular fibrosis [66]. More recently, same investigators showed reduction in miR-192 in renal cells in response to TGFβ1 [67]. Interestingly, Putta et al recently showed amelioration of renal fibrosis by administration of anti-miR-192 in a mouse model of diabetes, which involves TGFβ action [68]. Several other miRNAs including miR-93, miR-29, miR-214, miR-377 and mir-200 have been shown to play important role in TGFβ-driven renal fibrosis [13], [36], [40], [69], [70], [71].

Role of the miRNA miR-21 in various diseases including fibrosis has been extensively studied. A recent study has shown increased expression of miR-21 in patients with acute kidney injury and chronic allograft dysfunction both of which exhibit fibrosis of the kidney [72]. Furthermore, positive and negative role of miR-21 has been reported in cardiomyocyte hypertrophy *in vitro* [73], [74]. Moreover, tissue-specific action of miR-21 on PTEN expression is also reported. For example, miR-21 suppresses PTEN 3′UTR in hepatic cancer cells. In contrast, it did not inhibit PTEN 3′UTR in COS cells [45], [75]. However, in renal mesangial cells we demonstrate suppression of PTEN 3′UTR-driven reporter activity by miR-21, which regulates PTEN protein expression and its downstream Akt activation, resulting in phosphorylation of negative regulators of mTORC1 signaling (Figs. 1, 2, and 3).

miR-21 is significantly upregulated in rodent models of diabetic nephropathy, glomerulonephritis, ischemia reperfusion injury (IRI) and ureteral obstruction (UUO) [40], [41], [42], [72]. In IRI and UUO, increased expression of miR-21 was observed throughout the kidney especially in the glomerular cells. Less prominent expression was detected in the proximal epithelial compartment [72]. TGFβ is involved in pathologies observed in these models in which increased expression of miR-21 was evident in proximal tubular epithelial and glomerular mesangial cells [40], [41], [42], [72]. In concurrence, we found increased expression of miR-21 in human glomerular mesangial cells in response to TGFβ (Fig. S1). Although many direct targets of miR-21 exist, the specific proteins, which may mediate the effect of miR-21 on kidney fibrosis, have not been identified. Recently, Chau et al identified PPARα, as a significant target that mediates the fibrotic disease process in the kidney [72]. In the present study, we demonstrate another target of miR-21 in TGFβ-stimulated mesangial cells, PTEN, which regulates cellular hypertrophy and matrix protein expression.

TGFβ-induced mTORC1 regulates renal cell hypertrophy [23], [24], [25], [49], [50], [51]. Along with mTOR, this complex contains four more protein subunits: raptor, mLST8, deptor and PRAS40 [54], [57], [76]. All these proteins but mLST8 contribute to the activity of mTORC1 [57], [76], [77]. Nutrients and growth factors utilize independent mechanisms to activate mTORC1 [78]. For example, amino acids promote formation of GTP-bound Rag proteins. Rag-GTP binds to the lysozomal protein *Ragulator* to activate mTORC1 via binding to raptor [79], [80]. In growth factor stimulated cells, Akt kinase phosphorylates PRAS40, a raptor binding protein, which inhibits recruitment of substrate to the mTORC1 [54], [81]. Previously, it was shown that phosphorylated PRAS40 undergoes dissociation from this complex, resulting in increased mTORC1 activity [54], [55]. Similar to PRAS40 another inhibitor of mTORC1 is an upstream regulator tuberin. Tuberin heterodimerizes with hamartin and acts as a GTPase-activating protein for the mTORC1 activator Rheb [53]. Phosphorylation of tuberin by Akt kinase promotes its dissociation from hamartin, resulting in the formation of Rheb-GTP, which activates mTORC1 [53], [57], [76]. Results presented in Figure 3 show that miR-21 regulates phosphorylation of both tuberin and PRAS40 in response to TGFβ. Furthermore, we demonstrate that inhibition of miR-21 blocks TGFβ-stimulated activation of mTORC1 (Fig. 4). Thus our results provide a mechanism for TGFβ-induced activation of mTORC1 involving miR-21.

We have previously reported that PI 3 kinase-activated Akt controls TGFβ-stimulated hypertrophy and expression of plasminogen activator inhibitor-1, which contributes to the abundance of several matrix proteins in kidney tissues [23], [82]. In mesangial cells TGFβ-mediated expression of fibrotic proteins such as fibronectin and collagen is regulated by PI 3 kinase/Akt signal transduction and involves PTEN [22], [25], [83]. More recently Kato et al showed a role of PTEN in regulation of hypertrophy and, collagen and fibronectin by miR-216a [43]. In line with this observation, we now demonstrate a direct contribution of another miRNA, miR-21, which by targeting PTEN regulates mesangial cell hypertrophy and, expression of fibronectin and collagen in response to TGFβ (Figs. 5, 7A and 7B). In fact our data support the notion that miR-21 regulates expression of both these fibrotic proteins by downregulation of PTEN to activate Akt kinase (Fig. 7C and 7D).

The results from our laboratory and other have established a role of mTORC1 in cellular hypertrophy especially in renal cell hypertrophy [23], [24], [52], [84], [85]. Activation of mTORC1 involving miRNAs has been reported. Along with PRAS40 regulation of raptor, AMP-activated protein kinase (AMPK) phosphorylates raptor, resulting in inhibition of mTORC1 [86]. Binding of AMPK-phosphorylated raptor to 14-3-3ζ is necessary for inhibition of mTORC1 activity [86]. miR-451 directly targets 14-3-3ζ to induce unrestrained mTORC1 activity [87]. Additionally, miR-451 reduces the levels of AMPK-activating LKB1 kinase cofactor CAB39 (calcium binding protein 39) by binding to the 3′UTR of its mRNA [88], [89]. Similarly, miR-17-92 cluster-coded miR-19 directly targets the AMPK α1 catalytic subunit to inhibit its activity, thus promotes mTORC1 activation [90]. More recently miR-221 has been shown to downregulate REDD1, which activates tuberin by dissociating it from 14-3-3 and thus inhibiting mTORC1 activity [91], [92]. In many cancer cells, downregulation of miR-100, miR-101 and miR-199-3p, which bind to the 3′UTR of mTOR mRNA to block its protein levels, has been shown [93], [94], [95]. However, the role of these miRNAs in kidney cells and in the setting of renal fibrosis has not been investigated. We present data showing involvement of the miRNA miR-21 in activation of mTORC1 in response to the fibrotic cytokine TGFβ (Fig. 4). Our results demonstrate that TGFβ-stimulated miR-21 regulates hypertrophy of mesangial cells by targeting PTEN via activation of mTORC1 (Fig. 6). Furthermore, our results demonstrate a role of miR-21 targeted mTORC1 in TGFβ-stimulated expression of both fibronectin and collagen (Fig. 8).

Although administration of anti-miR-21 has been shown to block fibrosis in renal tissues, whether it derepresses any specific target protein has not been examined [41], [72]. Confirmation of derepression of target proteins such as PTEN in the miR-21-treated animal models of fibrosis will be necessary to establish the specificity of the therapy. Furthermore, it is important to establish a reciprocal correlation between expression of miR-21 and PTEN in renal tissues of patients with renal fibrosis. It will be beneficial if this correlation can be detected in subjects before the onset of the disease such as in prediabetics.

## Materials and Methods

### Materials

Recombinant TGFβ1 was purchased from R & D, Minneapolis, MN. Protease inhibitor cocktail, phenylmethylsulfonylfluoride, NP-40, Na_3_VO_4_, and fibronectin and β-actin antibody were obtained from Sigma, St Louis, MO. Phospho-Akt (Ser473), phospho-Akt (thr-308), Akt, phospho-S6 kinase, S6 kinase, phospho-4EBP-1 (Thr-37/46) phospho-4EBP-1 (Ser-65) 4EBP-1, phospho-GSK3β, GSK3β, phospho-tuberin (Thr 1462), tuberin, phospho-PRAS40 (Thr 246), PRAS40, phospho-mTOR (Ser-2448) and mTOR antibodies were purchased from Cell Signaling, Boston, MA. siRNA pool of three oligonucleotides against PTEN mRNA, collagen II (α2) and PTEN antibodies were obtained from Santacruz, Delaware, CA. RT^2^ real-time SYBR green/ROX PCR mix, RT^2^ miRNA first strand synthesis kit, primers for detecting mature miR-21 and GAPDH primers were purchased from Superarray, Frederick, MD. U6 primers for normalization of miR-21 expression were obtained from Ambion, Austin, TX. ^35^S-methione was purchased from PerkinElmer, Boston, MA. FuGene HD transfection reagent was purchased from Roche Molecular Biology, Indianapolis, IN. TRIZol reagent for RNA preparation was obtained from InVitrogen, Carlsbad CA. Luciferase reporter assay kit was purchased from Promega, Madison, WI. CMV-miR-21 expression plasmid was a gift from Dr. A. Hata, Tufts University School of Medicine, Boston, MA [96]. PTEN 3′UTR-Luc reporter plasmid was provided by Dr. T. Patel, Ohio University [45]. miR-21 Sponge plasmid was kindly provided by Dr. P. A. Sharp, MIT, Boston [46]. Constitutively active mTOR expression plasmid was provided by Dr. Tatsuya Maeda, The University of Tokyo, Japan and has been described previously.

### Cell Culture and Transfection

Preparation of human renal glomerular mesangial cells were described previously [97]. Frozen cells were thawed and grown in DMEM with 10% fetal bovine serum essentially as described previously [98], [99], [100]. The cells were used between passages 8 and 12. The cells were transfected with indicated plasmids in 12-well or 24 well culture plates using Fugene HD transfection reagent as described previously [23], [55], [62], [82].

### Cell Lysis and Immunoblotting

For each experiment, cells were washed 2x with PBS and radioimmunoprecipitation buffer (20 mM Tris-HCl, pH 7.5, 5 mM EDTA, 150 mM NaCl, 1% NP-40, 1 mM Na_3_VO_4_, 1 mM PMSF and 0.1% protease inhibitor cocktail) was added. The cell monolayer was incubated at 4°C for 30 minutes. The monolayer was scraped and centrifuged for 20 minutes at 4°C. The supernatant was collected and protein was estimated. Equal amounts of cell lysates were separated by SDS polyacrylamide gel electrophoresis. The separated proteins were transferred to PVDF membrane. Immunoblotting was carried out using indicated antibodies. The protein bands were developed using HRP-conjugated secondary antibodies with ECL chemiluminiscent reagent as described previously [23], [55], [62], [82].

### Real Time Quantitative RT-PCR

Total RNA was prepared using TRIZol reagent as described [101]. First strand cDNA was synthesized by RT^2^ kit according to the instruction provided by the vendor. qRT-PCR was performed in real-time PCR machine (7500, Applied Biosystems). U6 primers were used for normalization. Each sample was analyzed in duplicate. PCR conditions were as follows: 94°C for 10 minutes, followed by 40 cycles at 94°C for 30 seconds, 56°C for 30 seconds, 72°C for 30 seconds. Primers used for detection of pre-miR-21 were: Forward, 5′-TGTCGGGTAGCTTATCAGAC-3′; Reverse, 5′-TTCAGACAGCCCATCGACTG-3′.

### End Point RT-PCR

To determine the expression of miR-21 Sponge, we detected the levels of GFP mRNA as a surrogate [46]. One µg of total RNA from miR-21 Sponge-transected mesangial cells was reverse transcribed and amplified to detect GFP mRNA. PCR conditions were: 94°C for 10 minutes, followed by 40 cycles at 94°C for 30 seconds, 58°C for 30 seconds, 72°C for 30 seconds. The primers used for detection of GFP mRNA are as follows: Forward primer: 5′-ACGGCAAGCTGACCCTGAAG-3′; Reverse primer: 5′-GGGTGCTCAGGTAGTGGTTG-3′.

### Luciferase Activity

Lysates from reporter-transfected mesangial cells were used to measure luciferase activity using a kit as described previously [62], [82], [102]. The data are presented as mean of luciferase activity per microgram protein as arbitrary units ± SE of indicated measurements as described in the figure legends.

### Protein Synthesis and Hypertrophy

Transfected mesangial cells were serum-starved 16 hours followed by incubation with 2 ng/ml TGFβ for 24 hours. Protein synthesis was determined as ^35^S-methionine incorporation as described previously [25], [55]. For measurement of hypertrophy, after incubation, the cells were trypsinized and counted in the hemocytometer. The cells were then centrifuged at 4000xg for 5 minutes and the cell pellets were washed with PBS, lysed in RIPA buffer and protein content was measured. Hypertrophy was determined as increase in protein content per cell as described previously [23], [25], [55].

### Statistics

The data were analyzed by paired t-test. Where necessary the significance of the data was determined by ANOVA followed by Student-Newman-Keuls analysis as described previously [23], [25], [55], [62]. p value less than 0.05 was considered as significant.

## Supporting Information

Figure S1 **Expression of miR-21 in response to TGFβ in human mesangial cells.** Serum-starved mesangial cells were incubated with 2 ng/ml TGFβ for 24 hours. Total RNA from these cells were used for detection of Pre-miR-21 (panel A) and mature miR-21 (panel B) as described in the Materials and Methods. For panel A, mean ± SE of triplicate measurements is shown. *p = 0.0005 vs control. For panel B, mean ± SE of six measurements is shown. *p = 0.0001 vs control.
(TIF)

Figure S2 **Expression of CMV miR-21 for the results described in** **Figure 1B and 1C** **.** Mesangial cells were transfected with CMV miR-21 as described in the legend of **Figure 1B** and 1C. Total RNAs were used to detect mature miR-21 levels by qRT-PCR as described in the Materials and Methods.
(TIF)

Figure S3 **Expression of miR-21 Sponge for the results described in** **Figure 1D and 1E** **.** (A) Structure of the miR-21 Sponge plasmid. The sponge sequences are in the 3′ end of the GFP mRNA followed by poly (A) site under the control of cytomegalovirus promoter. (B and C) Expression of GFP as a measure of the Sponge expression. Total RNAs from miR-21 Sponge-transected cells described in **Figure 1D** and 1E were tested for GFP mRNA expression as described in the Materials and Methods. Expression of GAPDH was used as control.
(TIF)

Figure S4 **Expression of miR-21 Sponge for the results shown in** **Figure 2A–C** **.** Mesangial cells were transfected with miR-21 Sponge and treated with TGFβ as described in the legend of Figure 2A–C. Total RNAs were used to detect GFP and GAPDH as indicated.
(TIF)

Figure S5 **Expression of miR-21 Sponge for the results shown in** **Figure 3A and 3B** **.** Mesangial cells were transfected with miR-21 Sponge and treated with TGFβ as described in the legend of **Figure 3A** and 3B. Total RNAs were used to detect GFP and GAPDH as indicated.
(TIF)

Figure S6 **Expression of miR-21 Sponge for the results shown in** **Figure 4A–C** **.** Mesangial cells were transfected with miR-21 Sponge and treated with TGFβ as described in the legend of Figure 4A–C. Total RNAs were used to detect GFP and GAPDH as indicated.
(TIF)

Figure S7 **Expression of miR-21 Sponge for the results shown in** **Figure 5A–D** **.** Mesangial cells were transfected with miR-21 Sponge and siPTEN (panels A and B) or miR-21 Sponge plus Gag-Akt and treated with TGFβ as described in the legend of Figure 5A–D. Total RNAs were used to detect GFP and GAPDH as indicated.
(TIF)

Figure S8 **Expression of miR-21 Sponge for the results shown in** **Figure 6A and 6B** **.** Mesangial cells were transfected with miR-21 Sponge and CA mTOR and treated with TGFβ as described in the legend of **Figure 6A** and 6B. Total RNAs were used to detect GFP and GAPDH as indicated.
(TIF)

Figure S9 **Expression of miR-21 Sponge for the results shown in** **Figure 7A–D** **.** Mesangial cells were transfected with miR-21 Sponge and siPTEN (panels A and B) or miR-21 Sponge plus Gag-Akt and treated with TGFβ as described in the legend of Figure 7A–D. Total RNAs were used to detect GFP and GAPDH as indicated.
(TIF)

Figure S10 **Expression of miR-21 Sponge for the results shown in** **Figure 8A and 8B** **.** Mesangial cells were transfected with miR-21 Sponge and CA mTOR and treated with TGFβ as described in the legend of **Figure 8A** and 8B. Total RNAs were used to detect GFP and GAPDH as indicated.
(TIF)
