# Supplementary figures and images for: TGFβ-Stimulated MicroRNA-21 Utilizes PTEN to Orchestrate AKT/mTORC1 Signaling for Mesangial Cell Hypertrophy and Matrix Expansion
Source: PLoS One. 2012 Aug 3;7(8):e42316. doi: 10.1371/journal.pone.0042316 (PMC3411779; doi:10.1371/journal.pone.0042316)

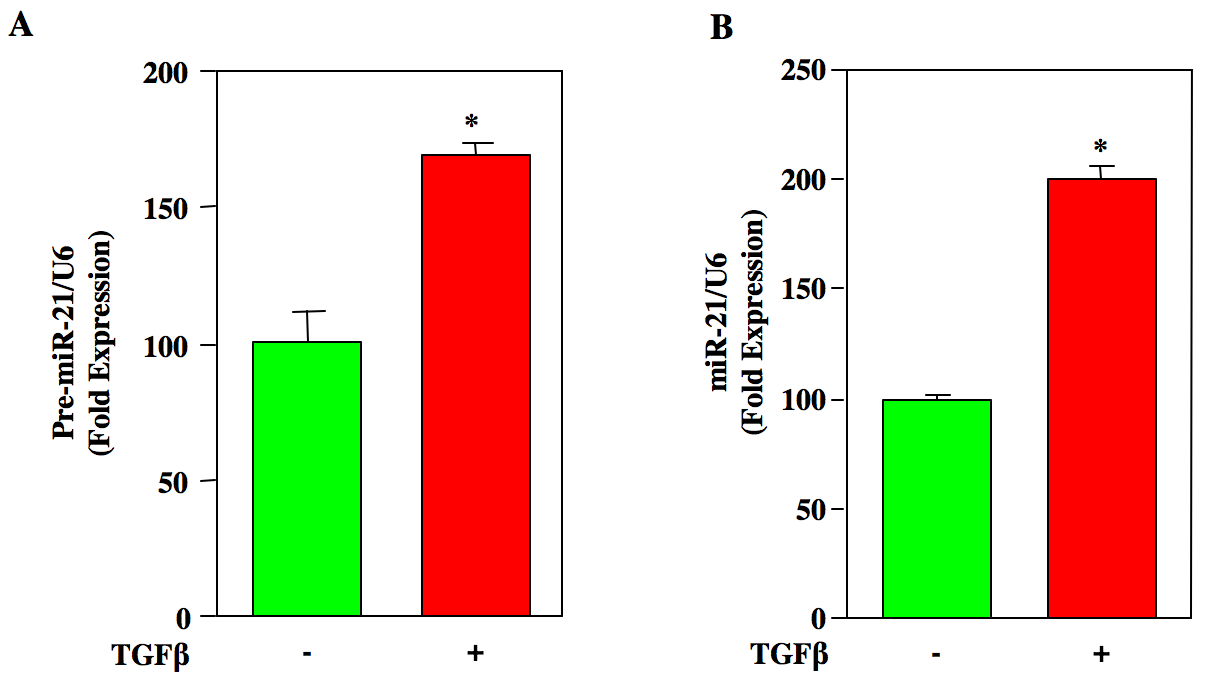

Supplement: Figure S1 — Expression of miR-21 in response to TGFβ in human mesangial cells. Serum-starved mesangial cells were incubated with 2 ng/ml TGFβ for 24 hours. Total RNA from these cells were used for detection of Pre-miR-21 (panel A) and mature miR-21 (panel B) as described in the Materials and Methods. For panel A, mean ± SE of triplicate measurements is shown. *p = 0.0005 vs control. For panel B, mean ± SE of six measurements is shown. *p = 0.0001 vs control. (TIF) [file pone.0042316.s001.tif]

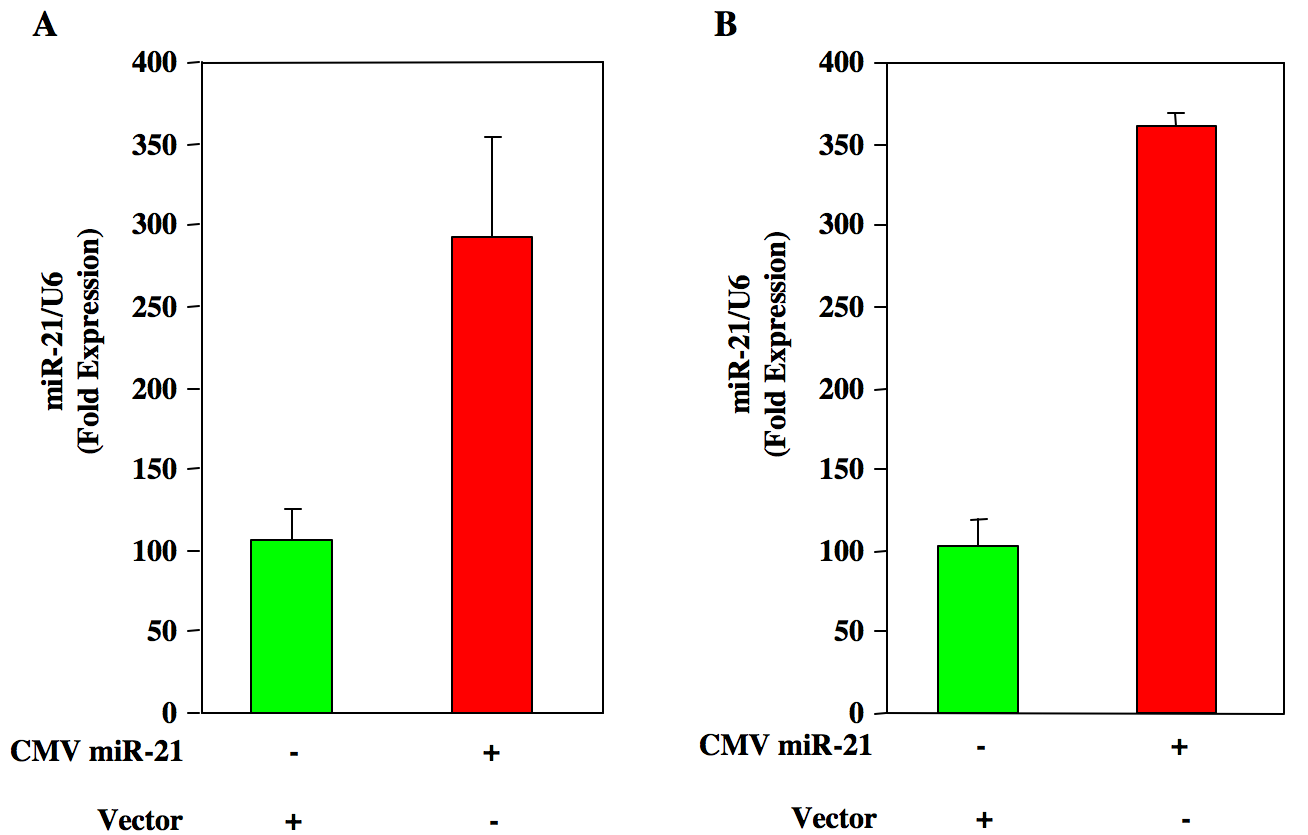

Supplement: Figure S2 — Expression of CMV miR-21 for the results described in Figure 1B and 1C . Mesangial cells were transfected with CMV miR-21 as described in the legend of Figure 1B and 1C. Total RNAs were used to detect mature miR-21 levels by qRT-PCR as described in the Materials and Methods. (TIF) [file pone.0042316.s002.tif]

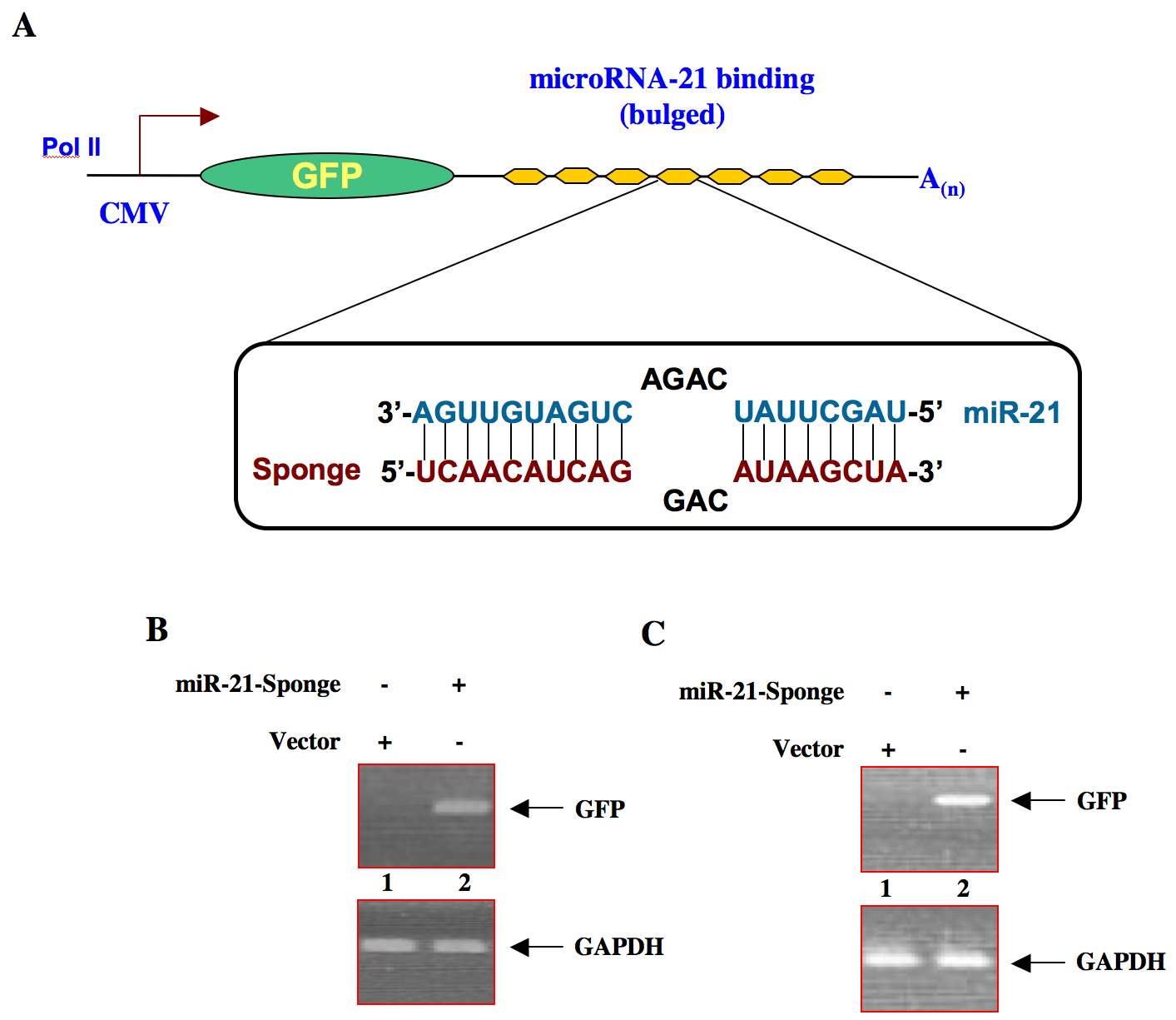

Supplement: Figure S3 — Expression of miR-21 Sponge for the results described in Figure 1D and 1E . (A) Structure of the miR-21 Sponge plasmid. The sponge sequences are in the 3′ end of the GFP mRNA followed by poly (A) site under the control of cytomegalovirus promoter. (B and C) Expression of GFP as a measure of the Sponge expression. Total RNAs from miR-21 Sponge-transected cells described in Figure 1D and 1E were tested for GFP mRNA expression as described in the Materials and Methods. Expression of GAPDH was used as control. (TIF) [file pone.0042316.s003.tif]

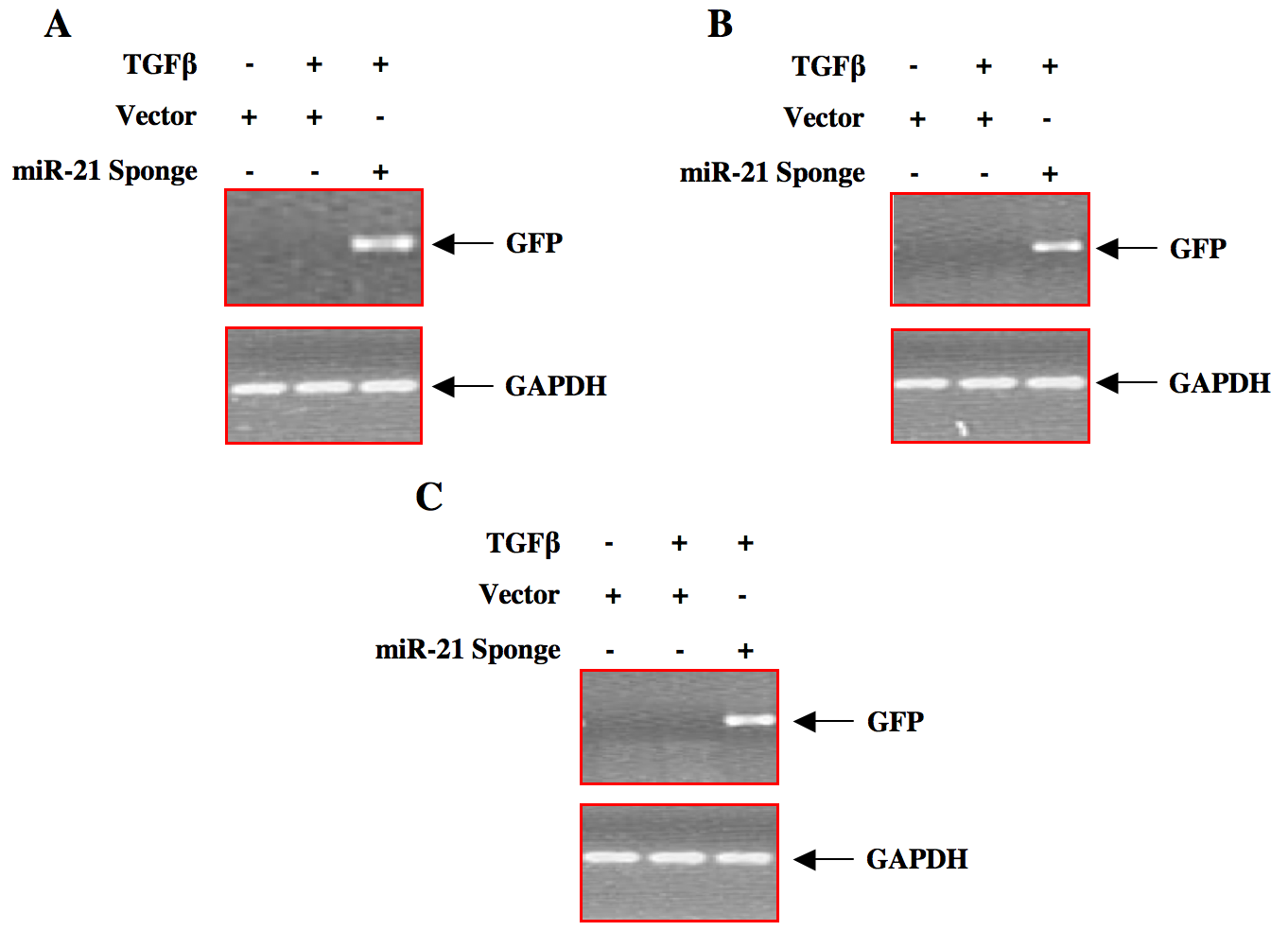

Supplement: Figure S4 — Expression of miR-21 Sponge for the results shown in Figure 2A–C . Mesangial cells were transfected with miR-21 Sponge and treated with TGFβ as described in the legend of Figure 2A–C. Total RNAs were used to detect GFP and GAPDH as indicated. (TIF) [file pone.0042316.s004.tif]

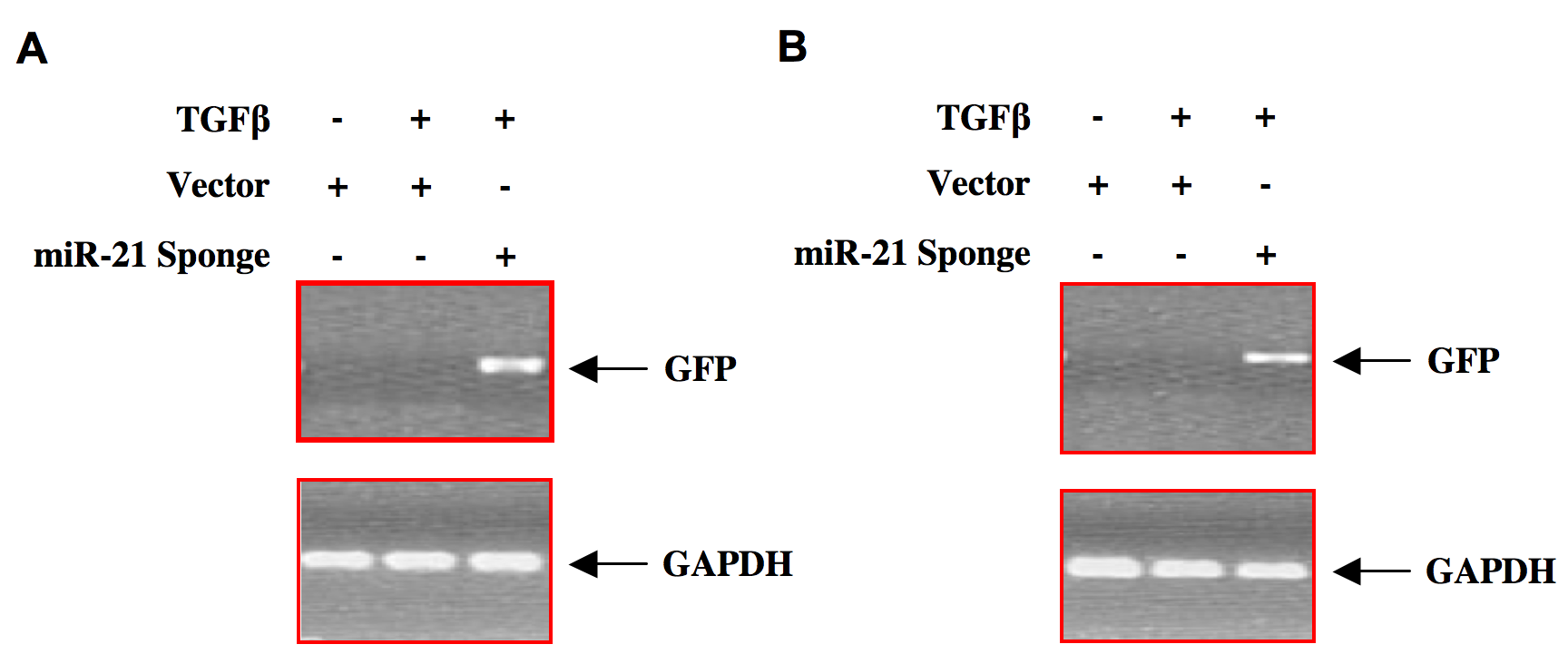

Supplement: Figure S5 — Expression of miR-21 Sponge for the results shown in Figure 3A and 3B . Mesangial cells were transfected with miR-21 Sponge and treated with TGFβ as described in the legend of Figure 3A and 3B. Total RNAs were used to detect GFP and GAPDH as indicated. (TIF) [file pone.0042316.s005.tif]

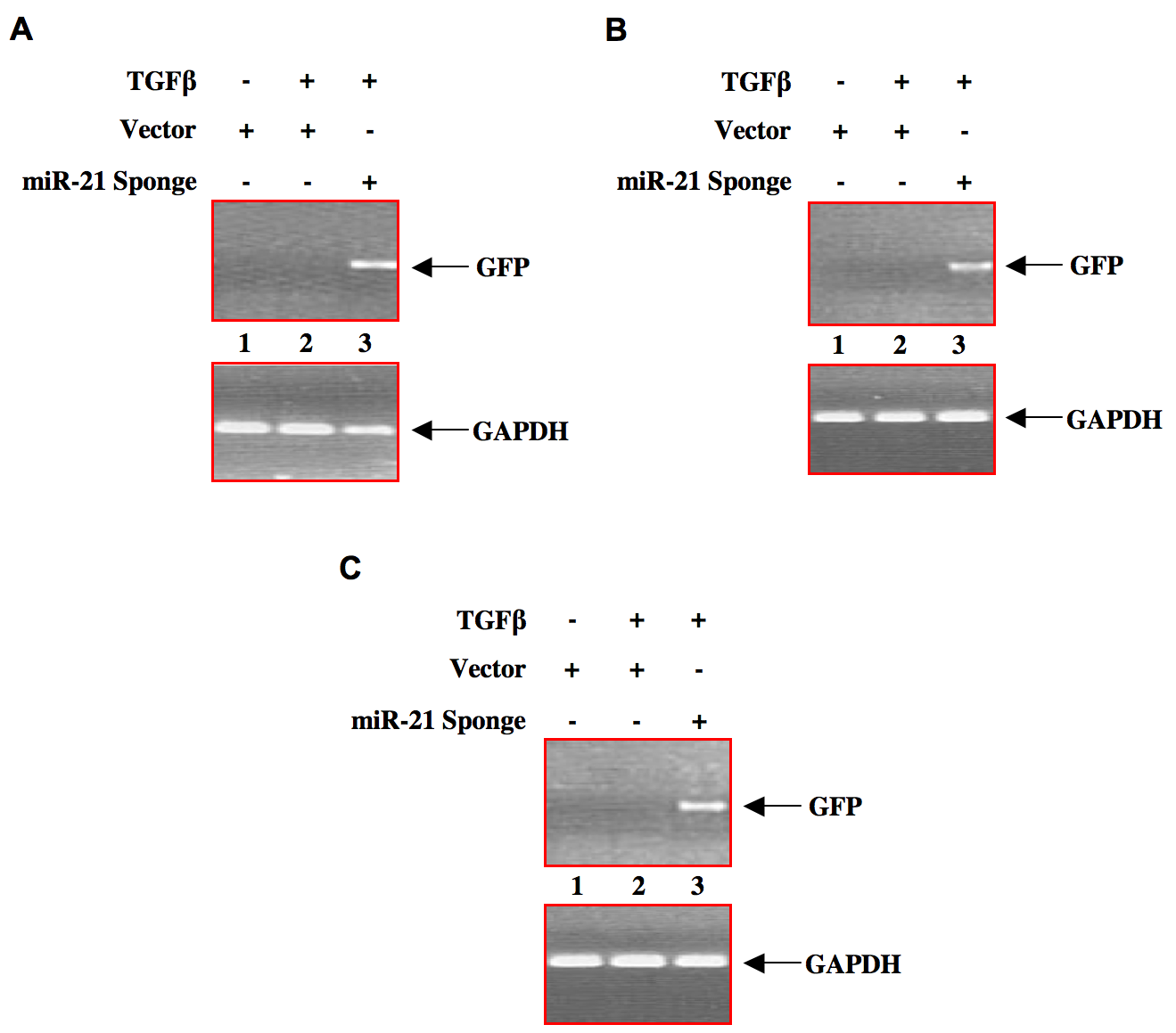

Supplement: Figure S6 — Expression of miR-21 Sponge for the results shown in Figure 4A–C . Mesangial cells were transfected with miR-21 Sponge and treated with TGFβ as described in the legend of Figure 4A–C. Total RNAs were used to detect GFP and GAPDH as indicated. (TIF) [file pone.0042316.s006.tif]

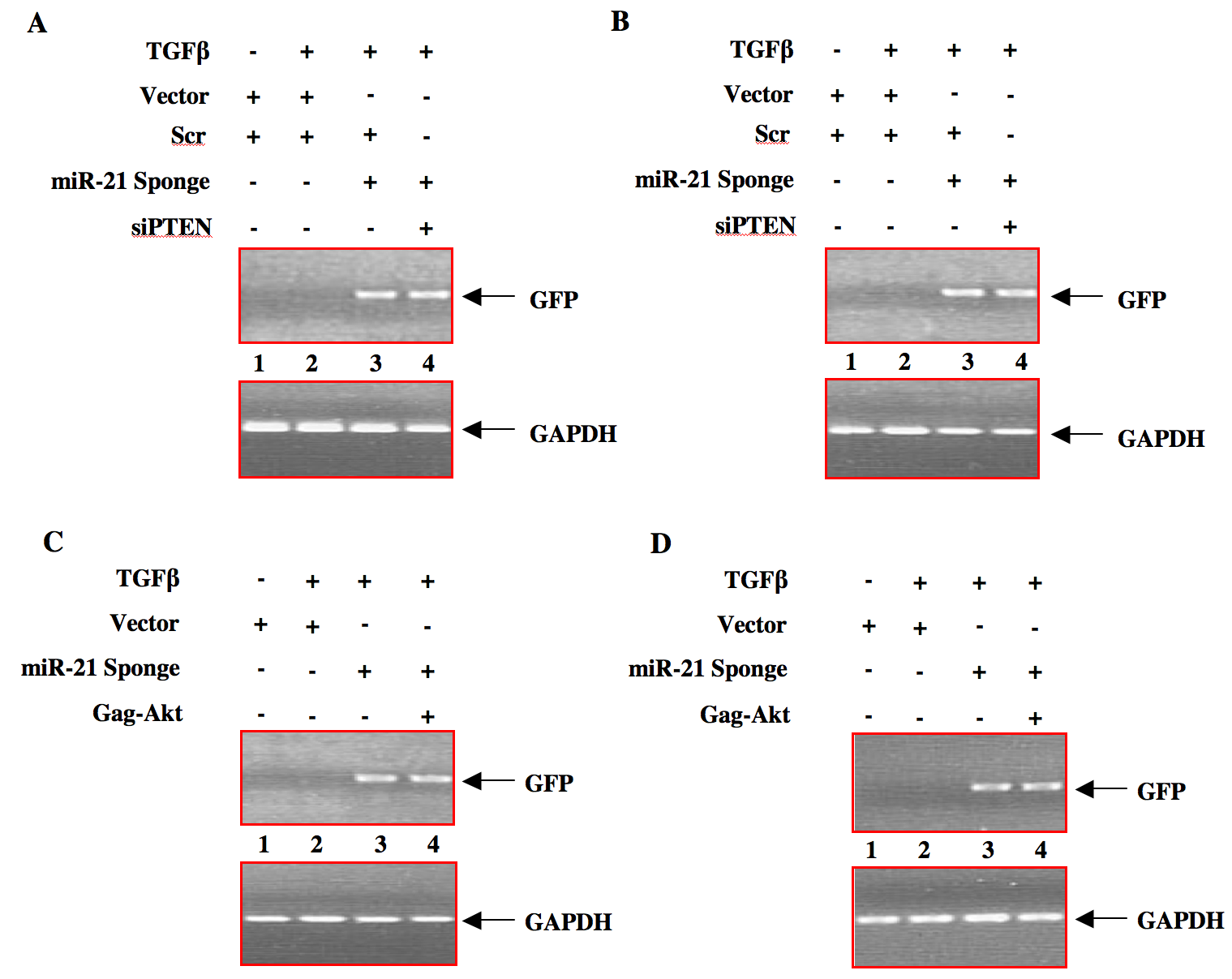

Supplement: Figure S7 — Expression of miR-21 Sponge for the results shown in Figure 5A–D . Mesangial cells were transfected with miR-21 Sponge and siPTEN (panels A and B) or miR-21 Sponge plus Gag-Akt and treated with TGFβ as described in the legend of Figure 5A–D. Total RNAs were used to detect GFP and GAPDH as indicated. (TIF) [file pone.0042316.s007.tif]

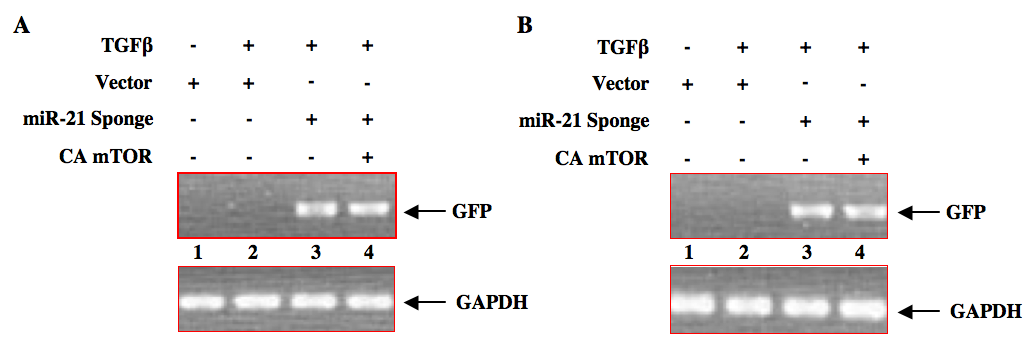

Supplement: Figure S8 — Expression of miR-21 Sponge for the results shown in Figure 6A and 6B . Mesangial cells were transfected with miR-21 Sponge and CA mTOR and treated with TGFβ as described in the legend of Figure 6A and 6B. Total RNAs were used to detect GFP and GAPDH as indicated. (TIF) [file pone.0042316.s008.tif]

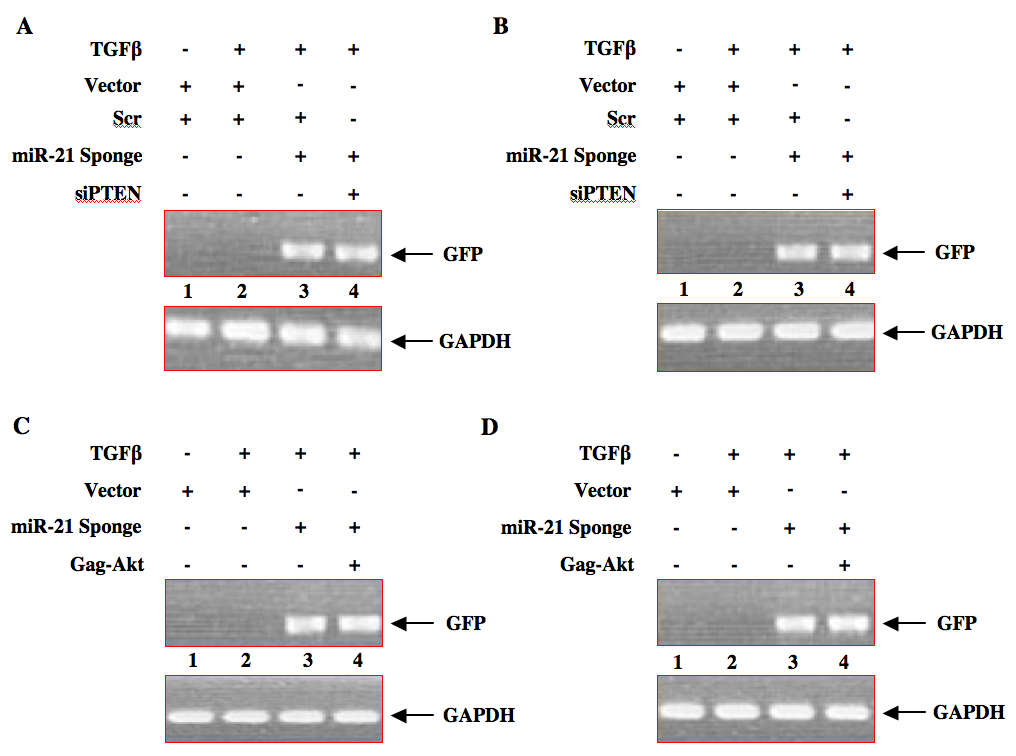

Supplement: Figure S9 — Expression of miR-21 Sponge for the results shown in Figure 7A–D . Mesangial cells were transfected with miR-21 Sponge and siPTEN (panels A and B) or miR-21 Sponge plus Gag-Akt and treated with TGFβ as described in the legend of Figure 7A–D. Total RNAs were used to detect GFP and GAPDH as indicated. (TIF) [file pone.0042316.s009.tif]

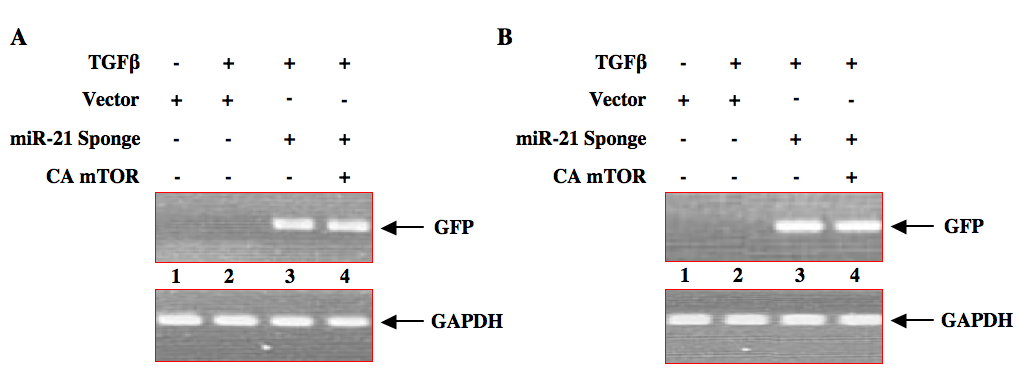

Supplement: Figure S10 — Expression of miR-21 Sponge for the results shown in Figure 8A and 8B . Mesangial cells were transfected with miR-21 Sponge and CA mTOR and treated with TGFβ as described in the legend of Figure 8A and 8B. Total RNAs were used to detect GFP and GAPDH as indicated. (TIF) [file pone.0042316.s010.tif]
